# Cognitive and affective Theory of Mind in adolescence: developmental aspects and associated neuropsychological variables

**DOI:** 10.1007/s00426-019-01263-6

**Published:** 2019-11-08

**Authors:** Edith Theresa Gabriel, Raphaela Oberger, Michaela Schmoeger, Matthias Deckert, Stefanie Vockh, Eduard Auff, Ulrike Willinger

**Affiliations:** Department of Neurology, Medical University of Vienna, Waehringer Guertel 18-20, 1090 Vienna, Austria

## Abstract

**Abstract:**

Theory of Mind (ToM) is the ability to represent and attribute mental states to oneself and others. So far, research regarding ToM processing across adolescence is scarce. Existing studies either yield inconsistent results or did not or not thoroughly investigate aspects like higher order ToM and associated neuropsychological variables which the current study tried to address. 643 typically developing early, middle, and late adolescents (age groups 13–14; 15–16; 17–18) performed cognitive and affective ToM tasks as well as neuropsychological tasks tapping the cognitive or affective domain. Regarding both ToM types, 15- to 16-year-olds and 17- to 18-year-olds outperformed 13- to 14-year-olds, whereas females were superior regarding cognitive ToM. Across adolescence, cognitive and affective ToM correlated with attention and affective intelligence, whereas working memory, language comprehension, and figural intelligence additionally correlated with cognitive ToM. In early adolescence, attention correlated with both ToM types, whereas cognitive ToM further correlated with language comprehension and affective ToM with verbal intelligence, verbal fluency, and verbal flexibility. In middle and late adolescence, affective intelligence correlated with both ToM types, whereas cognitive ToM additionally correlated with working memory, language comprehension, and figural intelligence. The current study shows a developmental step regarding cognitive and affective ToM in middle adolescence as well as gender differences in cognitive ToM processing. Associations between neuropsychological variables and ToM processing were shown across adolescence and within age groups. Results give new insights into social cognition in adolescence and are well supported by neuroscientific and neurobiological studies regarding ToM and the integration of cognitive and affective processes.

**Graphic abstract:**

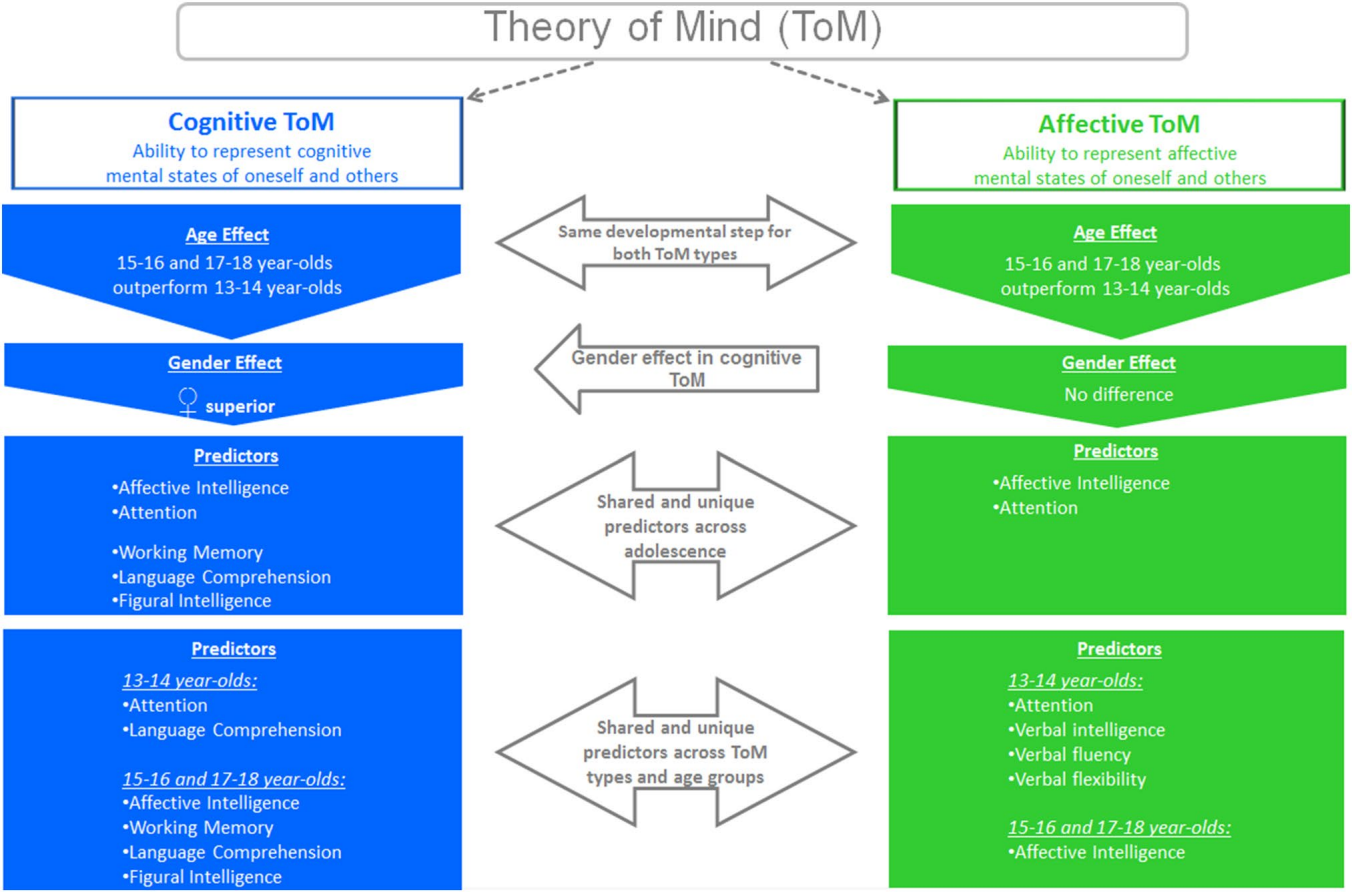

## Research highlights

- Cognitive and affective Theory of Mind (ToM) abilities increase significantly in adolescence and a developmental step in middle adolescence can be seen.
- Girls outperform boys regarding cognitive ToM, but not regarding affective ToM.
- Across adolescence, attention and affective intelligence are associated with cognitive and affective ToM; Cognitive ToM is additionally predicted by working memory, language comprehension, and figural intelligence.
- Associated neuropsychological variables vary across ToM type and adolescent age group.
- Results are supported by neuroscientific and neurobiological studies regarding ToM and the integration of cognitive and affective processes.

## Introduction

### Theory of Mind (ToM): definition, components, and its neurobiological bases

“Theory of Mind” (ToM), first introduced by Premack and Woodruff (1978), is the ability to represent and attribute mental states such as knowledge, beliefs, expectations, intentions, and emotions to oneself and others, and can be used to understand and predict one’s own and other’s behavior (e.g., Schlaffke et al., 2015). ToM is a complex construct that can be divided into affective ToM and cognitive ToM (e.g., Shamay-Tsoory & Aharon-Peretz, 2007). Affective ToM is represented by implications about emotions whereas cognitive ToM involves implications about knowledge, intentions, and beliefs (e.g., O’Brien et al., 2011). ToM seems to be a multi-order construct involving levels of increasing complexity ranging from a rather basic and simple first-order ToM (e.g., “X thinks or feels …”), to a second-order ToM (e.g., “X thinks that Y feels …”), and the more advanced and complex third-order ToM (e.g., “X believes that Y assumes that Z intends …”), see for example Brune and Brune-Cohrs (2006), Perner and Wimmer (1985), or Wimmer and Perner (1983).

A specific neuronal ToM network was identified involving, inter alia, prefrontal, cingulate, temporal, parietal, limbic, and other subcortical regions (see e.g., Abu-Akel & Shamay-Tsoory, 2011). In this context, Abu-Akel and Shamay-Tsoory (2011) postulated a novel neurobiological model of ToM that indicates different processing steps in different ToM-specific brain regions and how they are influenced by attention and neurochemical systems. Representations of both cognitive and affective mental states are formed at the temporoparietal junction (TPJ) and subsequently pass through the superior temporal sulcus (STS) or the precuneus/posterior cingulate complex (PCun/PCC) to different limbic–paralimbic regions where the cognitive or affective values are determined. Cognitive mental states are enabled by the dorsal regions of the temporal pole (dTP), anterior cingulate cortex (dACC), medial prefrontal cortex (dmPFC), and lateral prefrontal cortex (dlPFC) whereas affective mental states are enabled by the ventral striatum, amygdala, ventral temporal pole (vTP), ventral anterior cingulate cortex (vACC), the orbitofrontal cortex (OFC), the ventral medial prefrontal cortex (vmPFC), and inferolateral frontal cortex (ilFC). The interaction of cognitive and affective networks is seemingly mediated within the ACC. Whereas ToM performance is based on a network of distinct brain regions, the activation of this network seems to be significantly influenced by dorsal and ventral attention and selection systems as well as dopaminergic and serotonergic systems. Whereas this neurobiological model refers to mentalizing brain circuits in the brain of adults, according to the authors it further seems to provide a “*suitable framework for examining the development of ToM*” (Abu-Akel & Shamay-Tsoory, 2011, page 2981).

### ToM in childhood and adolescence: developmental aspects

The ability to understand another person’s beliefs and reactions (1st order ToM) emerges approximately at age four to five (Wimmer & Perner, 1983). Whilst 6- and 7-year-olds usually are able to mentally represent and understand second-order beliefs (Perner & Wimmer, 1985), it has been shown that the ability to deal with third-order representations starts rudimentally when children are approximately 7 years (Astington & Dack, 2008). The development of ToM does not end in childhood but at least continues throughout adolescence and young adulthood (e.g., Vetter, Altgassen, Phillips, Mahy, & Kliegel, 2013).

Findings regarding cognitive ToM during adolescence, late adolescence, and emerging adulthood are not consistent. Whilst some studies show superiority of emerging adults (Altgassen, Vetter, Phillips, Akgun, & Kliegel, 2014), other studies show no significant differences between adolescence and adulthood (e.g., Sebastian et al., 2012). To date, research regarding the development of higher order cognitive ToM in adolescence is scarce. In this context, Valle, Massaro, Castelli and Marchetti (2015), for example, show that emerging adults outperform adolescents in third-order tasks while no differences can be found within adolescence. Regarding these results, methodological issues arise as the authors pick only two age-groups (14 and 17 years) in order to represent young and late adolescents. Affective ToM research, on the other hand, yields inconsistent results as some studies show that adolescents perform worse than emerging adults (Sebastian et al., 2012), whereas other studies find no differences (Vetter, Weigelt, Dohnel, Smolka, & Kliegel, 2014). With respect to gender differences, studies suggest that girls show greater social awareness than boys (Bosco, Gabbatore, & Tirassa, 2014), whereas no gender differences regarding affective ToM could be found (Vetter et al., 2013; Frank, Baron-Cohen, & Ganzel, 2015).

Across childhood and adolescence, great neurodevelopmental changes take place (e.g., Brain Development Cooperative Group, 2012). In this age span, not only is the brain reorganized drastically involving structural and functional development (e.g., Brain Development Cooperative Group, 2012; Lebel & Beaulieu, 2011; Sato et al., 2014; Shaw et al., 2008), but also there are changes in neurotransmitter systems (see e.g., Steinberg, 2016).

In the light of the previously mentioned neurobiological model of ToM (Abu-Akel & Shamay-Tsoory, 2011), especially in early, middle, and late adolescence, specific changes with respect to ToM-specific regions can be noted. In this context, approximately between ages 13 and 18, specific developments can be seen with respect to whole brain gray and white matter volume (Brain Development Cooperative Group, 2012), hippocampal regions and the amygdala (Hu, Pruessner, Coupé, & Collins, 2013; Krogsrud et al., 2014), temporal regions (e.g., Shaw et al., 2008), cingulate cortex (Shaw et al., 2008), subcortical regions (Brain Development Cooperative Group, 2012) as well as an ongoing re-organization in the prefrontal cortex (e.g., Blakemore, 2008; Shaw et al., 2008). Besides a general increase in brain connectivity (e.g., Sato et al., 2014), changes in ToM-specific connectivity between prefrontal, temporal, and temporo-parietal regions (Blakemore, 2008) were shown. Furthermore, great changes with respect to the serotonergic as well as the dopaminergic neurotransmitter system were shown in this age span (e.g., Steinberg, 2016).

### Neuropsychological variables associated with ToM^1^

Attention has been shown to be a predictor of children’s cognitive ToM (Bloom & German, 2000). In this context, poor cognitive and affective ToM skills in childhood and adolescence were shown to be related to poor attention (Austin, Groppe, & Elsner, 2014). Furthermore, there seem to be anatomical as well as functional overlaps between mentalizing and attentional system networks, especially within the TPJ and ACC regions whereas these networks still evolve throughout adolescence (e.g., Abu-Akel & Shamay-Tsoory, 2011; Koziol, Joyce, & Wurglitz, 2014).

Working memory is strongly associated with cognitive and affective ToM (e.g., Amadó, Serrat, & Vallès-Majoral, 2016) as ToM development depends on a person’s growing ability to keep information in mind which allows for a fair judgment of another person’s mental state (Olson, 1993). Working memory activates a network of brain regions which partially overlaps with the ToM network such as the prefrontal cortex, the basal ganglia, and the anterior cingulate cortex (e.g., Brahmbhatt, McAuley, & Barch, 2008; Eriksson, Vogel, Lansner, Bergström, & Nyberg, 2015). A prolonged development of WM functions and changing underlying neuronal bases across childhood, adolescence, and adulthood was shown (e.g., Conklin, Luciana, Hooper, & Yarger, 2007; Sowell et al., 1999; Vogan, Morgan, Powell, Smith, & Taylor, 2016).

Verbal abilities, especially language comprehension, seem to predict cognitive (Ahmed & Miller, 2011; Astington & Jenkins, 1999; Frank, Baron-Cohen, & Ganzel, 2015) and affective ToM (Ahmed & Miller, 2011; Frank et al., 2015; Vetter et al., 2013) in childhood and (young) adulthood whereas verbal fluency seemingly influences both ToM types (Ahmed & Miller, 2011). Pragmatic language (Frank et al., 2015) and syntax (Astington & Jenkins, 1999) are predictive of cognitive ToM performance whilst affective ToM performance may require a basic understanding of emotion words (Ahmed & Miller, 2011). These findings are supported by shared regions of ToM and language processing networks like for example the temporal lobes (e.g., Abu-Akel & Shamay-Tsoory, 2011; Szaflarski et al., 2012).

Regarding the role of cognitive intelligence, inconsistent results can be found as some studies show a greater association of ToM with fluid intelligence rather than with crystallized intelligence (Maylor, Moulson, Muncer, & Taylor, 2002) whilst other studies show that ToM performance is equally related to both types of intelligence (Sullivan & Ruffman, 2004). In their meta-analysis, Baker, Peterson, Pulos and Kirkland (2014) showed a relation between intelligence and ToM performance whereas there was no difference between verbal and performance intelligence. These findings are in line with studies that show that ToM and reasoning processing show shared neural activity such as in the prefrontal cortex (e.g., Abu-Akel & Shamay-Tsoory, 2011; Donoso, Collins, & Koechlin, 2014). This is further supported by developmental aspects regarding this region (e.g., Blakemore, 2008).

Marked improvements in executive functions and ToM take place between ages three and six. For preschool children (4- and 5-year-olds, Carlson, Moses, & Breton, 2002), positive correlations between executive functions and cognitive ToM were shown whereas for 6- to 12-year-olds positive correlations between executive functions and cognitive and affective ToM could be found (Austin et al., 2014). Furthermore, it was shown that in early adolescence cognitive and affective ToM performance is associated with executive function performance (Im-Bolter, Agostino, & Owens-Jaffray, 2016) whereas for adolescence and young adulthood executive functions were shown to be predictive of affective ToM (Vetter et al., 2013). In this context, shared neuronal correlates where shown between executive functions and ToM processing like for example in the prefrontal cortex (see e.g., Blakemore, 2008).

In their model, Mayer and Salovey (1997) define affective intelligence as follows: it “involves the ability to perceive accurately, appraise, and express emotion; the ability to access and/or generate feelings when facilitate though; the ability to understand emotion and emotional knowledge; and the ability to regulate emotions to promote emotional and intellectual growth” (Mayer & Salovey, 1997, p. 5). The difference between affective intelligence and the concept of affective ToM is that affective ToM involves the ability to represent own as well as other’s feelings and emotional states (Schlaffke et al., 2015). Nevertheless, for both abilities overlapping activation in a number of brain regions could be found (Mier et al., 2010), whereas some of these regions are significantly stronger activated in affective ToM. Emotion recognition is an important aspect of affective intelligence (Mayer & Salovey, 1997) and was shown to be a meaningful predictor of ToM ability (in this paper affective intelligence will be used as a synonym for emotion recognition). A longitudinal study by O’Brien et al. (2011) shows that affective intelligence at age three predicts improvements in cognitive ToM in 3- to 4-year-olds whereas ToM performance does not predict affective intelligence. Therefore, children seem to understand emotions before they understand mental states. Furthermore, correlations between affective intelligence and ToM performance are seen more frequently in 4-year-olds. These results indicate a growing integration of these skills over time. With respect to affective intelligence, marked improvements can be seen between age 6 and 19 whereas females partially outperform males (Williams et al., 2009). The results indicate that the relation between affective intelligence and affective ToM is still given in adulthood (Mier et al., 2010). In their neurobiological model, Abu-Akel and Shamay-Tsoory (2011) show that affective and cognitive ToM exhibit overlapping activation in specific brain regions and are closely connected. This is supported by imaging studies that show strong connections between cognitive and emotional processing, e.g., Pessoa, 2008).

### Aims of this study

Although there are studies which focus on ToM development across childhood as well as on differences between children, adolescents, and adults with respect to ToM ability, only a few studies address ToM development across adolescence. This is surprising, given the previously mentioned neurodevelopmental changes especially in adolescents between 13 and 18, and with respect to the development of brain regions associated with ToM processing (e.g., Abu-Akel and Shamay-Tsoory, 2011). Furthermore, previous studies on ToM in adolescence yielded inconsistent results and did not thoroughly investigate higher order ToM. Therefore, the first aim of the current study was to investigate basic and higher order ToM processing in this age span and to provide missing behavioral data regarding possible developmental changes which are indirectly indicated by neurodevelopmental changes in this age span. The second aim was to investigate to which degree ToM performance can be explained by age effects and neuropsychological variables. To this end, neuropsychological variables were chosen based on their shared neuronal correlates with ToM processing (see Abu-Akel & Shamay-Tsoory, 2011) and behavioral studies addressing their relations with ToM processing. As these variables were either not or not sufficiently investigated in adolescence so far, it was investigated whether attention, working memory, cognitive intelligence, affective intelligence, executive functions, and language comprehension are also associated with ToM processing across adolescence. Furthermore, in order to give a more exhaustive view on the relation between neuropsychological variables and ToM processing in adolescence, additional analyses were conducted for age groups separately.

These aims lead to following hypotheses: an age-related increase as well as possible single or multiple developmental steps with respect to cognitive ToM (H1.1) as well as affective ToM (H1.2) can be identified in adolescence. Across all age groups, cognitive ToM will be significantly associated with age, attention, working memory, language comprehension, cognitive intelligence, affective intelligence, and executive functions (H1.3). Furthermore, cognitive ToM will be significantly associated with attention, working memory, language comprehension, cognitive intelligence, affective intelligence, and executive functions within age groups 13–14 years (H1.4), 15–16 years (H1.5), and 17–18 years (H1.6). Across all age groups, affective ToM will be significantly associated with age, attention, working memory, language comprehension, cognitive intelligence, affective intelligence, and executive functions (H1.7). Furthermore, affective ToM will be significantly associated with attention, working memory, language comprehension, cognitive intelligence, affective intelligence, and executive functions within age groups 13–14 years (H1.8), 15–16 years (H1.9), and 17–18 years (H1.10).

## Materials and methods

### Participants

A sample of 643 (58.6% female, 41.4% male) 13- to 18-year old (*M *= 14.85, SD= 1.88) early, middle, and late adolescents were recruited from 13 public secondary schools in Vienna and Lower Austria. Participants were divided into three age groups: 13- to 14-year-olds (3rd grade, *n* = 218 (33.9%), 55.5% female), 15- to 16-year-olds (5th grade, *n* = 205 (31.9%), 62% female) and 17- to 18-year-olds (7th grade, *n* = 220 (34.2%), 58.6% female). One hundred thirty-six (21.2%) subjects had no brothers or sisters, 299 (46.5%) subjects had one sibling, and 208 (32.3%) subjects had two or more siblings. Five hundred seventy-three (89.1%) participants were Austrian whereas 70 (10.9%) participants had different nationalities. Most of the participants (488, 75.9%) lived with their parents, 127 (19.8%) lived only with their mother and 28 (4.3%) participants lived with their father, their grandparents, or in different living situations. The Chi-square test showed neither significant differences with respect to gender (*χ*^2^ (1) = 3.43, *p *= 0.064) nor regarding gender and age (*χ*^2^ (2) = 1.81, *p* = 0.404). After receiving permission by the responsible subsection of the Austrian federal ministry of education as well as by the respective headmasters and headmistresses, letters of agreement signed by parents and participants were obtained. Exclusion criteria were a history of or present psychiatric or neurological disorders, or a lack of agreement of either the participants or their legal guardian. The study protocol was approved by the Institutional Review Board of the Medical University of Vienna and meets the ethical principles of the Code of Ethics of the World Medical Association (Declaration of Helsinki) as well as the APA ethical standards for human research. Written informed consent for participation and publication was obtained by every participant or legal guardian, respectively.

### Materials

#### Cognitive ToM and language comprehension

Cognitive Theory of Mind in terms of false belief reasoning was measured with the ToM Stories for Children and Adolescents, a modified version of the ToM Stories for Adults. The ToM Stories for Adults were developed in order to measure basic (1st order) as well as higher order (2nd and 3rd order) false belief reasoning by Willinger et al. (in preparation). The ToM Stories for Adults comprise six stories that were developed based on the famous “Maxi story” for the assessment of first-order false belief by Wimmer and Perner (1983) as well as the “ice-cream story” for the assessment of second-order false belief by Perner and Wimmer (1985). The ToM Stories for Adults combine first and second-order as well as third-order false belief reasoning within single stories whereby the newly developed third-order false belief task was based on the ideas regarding the second-order false belief items by Perner and Wimmer (1985).

The structure of all six stories can be described as follows: after certain activities, two protagonists A and B share the same knowledge regarding a certain state of affairs X. After the two protagonists get separated, either one of the protagonists or a third protagonist C causes an unexpected change of state of affairs X into a new state of affairs Y like for example removing a target object. At this point in the story, a first-order false belief question regarding the actual state of affairs is posed, like for example “Where will protagonist A search for the object?” Afterwards, the unknowing protagonist A with the false belief gets updated on the new state of affairs Y by protagonist C or a fourth subject D. At this point in the story, a second-order belief question is posed, like for example “Where does protagonist B think that protagonist A will search for the object?” Furthermore, two control questions (in order to assess whether the second-order belief was fully understood at this point) are posed. These control questions are preceded by movements in the story that enable these questions but do not change the state of affairs meaningfully. Afterwards, the knowing protagonist B or C who in the beginning changed the state of affairs X into Y gets updated on the knowledge of the previously unknowing protagonist A by the same person that previously informed protagonist A (protagonist C or subject D). At this point in the story, two comprehension questions are posed which assess whether the storyline was understood. Afterwards, it is described that protagonist A does not know the actual knowledge of the knowing protagonist B or C. At this point in the story, a third-order belief question is posed like for example “What does protagonist A think that protagonist B thinks that protagonist A thinks where the object is?”. Furthermore, two control questions involving a preceding movement in the story are posed. Afterwards, protagonist A gets updated on the actual knowledge of the knowing protagonist B or C. At the end of the story, four comprehension questions are posed.

For each false belief question, 2–4 multiple-choice answers are presented involving one right answer and one to three distractors. The control and the comprehension questions are either presented in the previously mentioned multiple-choice format or require a dichotomous answer (yes or no). The false belief questions could only be scored correctly if the question itself as well as the appendant control questions were answered correctly. Across all stories, the correct responses to the false belief questions are summed, yielding a first-order, second-order, third-order as well as a total ToM score. Due to the nature of the comprehension questions assessing the comprehension of a verbally presented story, the correct answers are summed to yield a language comprehension score.

The ToM Stories for Adults were previously validated (Willinger et al., in preparation., yielding correlations of *r* = 0.08 for first-order ToM, *r* = 0.37 for second-order ToM, *r* = 0.32 for third-order ToM as well as *r* = 0.47 for total ToM when compared to an established ToM task (Schlaffke et al., 2015) which represents another methodical approach by using picture stories. Regarding the relatively simple first-order ToM (see also e.g., Wimmer & Perner, 1983), a ceiling effect in terms of a low variance was shown which, besides the different methodical approaches of both ToM tasks, presumably explains the low correlation. The task was adapted for the use in children and adolescents comprising three stories with the same structure as in the adult’s version, but with a simplified language. The ToM stories for Children and Adolescents yielded a Cronbach’s alpha of 0.76 (Correlation between Item and Total score = 0.315). The previously mentioned ceiling effect regarding first-order ToM in the adults can also be seen in the version for children and adolescents (see current study, Table 1).

**Table 1:** Means and standard deviations for the variables *cognitive* and *affective ToM* as well as the neuropsychological variables in 13- to 14-year-olds, 15- to 16-year-olds, and 17- to 18-year-olds

Age, grade	Affective ToM	Cognitive ToM 1st order	Cognitive ToM 2nd order	Cognitive ToM 3rd order	Cognitive ToM Total score	Language comprehension	Verbal Int.	Numerical Int.	Figural Int.	Attention	Working memory	Verbal fluency RWT P	Verbal fluency RWT Animals	Verbal flexibility RWT HT	Verbal flexibility RWT Sport-Fruits	Affective Int.
13–14 3rd grade																
*M*	17.60	2.45	1.61	0.66	4.72	11.24	9.04	6.56	7.14	98.38	14.00	6.86	17.94	5.10	5.89	32.32
SD	3.52	0.70	0.97	0.92	1.93	3.36	3.48	2.55	2.71	29.70	2.85	2.96	4.33	1.50	1.29	4.64
15–16 5th grade																
*M*	19.00	2.57	1.95	1.15	5.66	11.87	11.33	7.76	8.51	126.85	15.61	8.04	19.19	6.02	6.64	32.98
SD	3.07	0.66	0.94	1.06	1.97	3.41	3.82	2.86	2.96	28.20	2.72	3.11	4.39	1.69	1.43	4.13
17–18 7th grade																
*M*	19.76	2.69	2.16	1.24	6.09	12.23	12.33	7.87	8.78	131.06	16.15	9.10	20.42	6.44	7.27	34.29
SD	2.92	0.54	0.88	1.07	1.94	3.72	4.03	2.94	3.49	33.65	3.34	3.27	4.84	1.77	1.62	4.67
Total																
*M*	18.79	2.57	1.91	1.01	5.49	11.78	10.90	7.39	8.14	118.69	15.25	8.00	19.19	5.85	6.60	33.20
SD	3.31	0.65	0.96	1.05	2.03	3.52	4.02	2.85	3.15	33.93	3.12	3.25	4.64	1.75	1.56	4.56

Total *N* = 643

*ToM* Theory of Mind, *Int.* intelligence score, *RWT* Regensburg word fluency test, *RWT P* RWT “p”-words, *RWT HT* RWT “h- and t-words”

#### Affective ToM

Affective ToM was assessed with the Reading the Mind in the Eyes Test (Baron-Cohen, Wheelwright, Hill, Raste, & Plumb, 2001) which measures the ability to perceive and recognize another person’s emotional state. The test consists of pictures which show a specific part of the human face, namely the eyes, the eye-brows as well as the upper half of the nose. Each picture is accompanied by four words that describe emotional states (e.g., irritated, sarcastic, worried, friendly). The task requires choosing one correct emotional state word that describes best what the person in the picture feels, thinks, or expresses whereas the other three words are distractors. The number of correct answers was chosen for the statistical analyses. Validation studies regarding this task showed test–retest reliabilities ranging between 0.630 and 0.833 (e.g., Fernández-Abascal, Cabello, Fernández-Berrocal, & Baron-Cohen, 2013; Pfaltz et al., 2013; Vellante et al., 2013).

#### Cognitive intelligence

Cognitive intelligence was assessed with subtests of the Intelligence Structure Test—Revised (I-S-T 2000R; Amthauer, Brocke, Liepmann, & Beauducel, 2001), an intelligence test battery which measures, besides others, verbal, numerical, and figural intelligence. Verbal intelligence was assessed with the subtests “verbal analogies” and “similarities” and measures processing of verbal material, reasoning, vocabulary, and building relations between terms. In the subtest “verbal analogies” (Cronbach alpha = 0.74), the participants are presented with three given terms, whereby there is a relation between the first two terms. This relation needs to be recognized and out of five given possible answers one term should be chosen that has a similar relation with the third given term. In the subtest “similarities” (Cronbach alpha = 0.76), out of six words, two have to be chosen which share a hypernym. Numerical intelligence was assessed with the subtests “number series” and “numerical signs” and measures building logical relations between numbers as well as numeracy. In the subtest “number series” (Cronbach alpha = 0.91), series of numbers are presented which are constructed according to specific rules. For each series, the rules need to be recognized and the next number inserted. In the subtest “numerical signs” (Cronbach alpha = 0.86), equations in the range of rational numbers are presented whereby the right answer is given but the arithmetic operators are missing. The participants need to solve the equations by inserting the right arithmetic operators of the four basic arithmetical operations. Figural intelligence was assessed with the subtests “figure selection” and “matrices” and measures processing of figural pictorial material involving two-dimensional figures, understanding proportions of areas, and building logical relations between figures. In the subtest “figure selection” (Cronbach alpha = 0.77), ten dissected figures are shown. Participants are asked to mentally put the pieces of each figure together and to note which of five answer figures each dissected figure shows. In the subtest “matrices” (Cronbach alpha = 0.71), figures arranged in rows and columns are shown whereby these arrangements are built according to certain rules. One figure of each arrangement is missing. The test requires identifying the rule and choosing the appropriate figure out of a set of choices. For each intelligence index, the correct answers of the respective two subtests were summed up, yielding verbal, numerical, and figural intelligence scores that were used in the analyses.

#### Affective intelligence

Affective intelligence was determined using the Facially Expressed Emotion Labeling (FEEL) (Kessler, Bayerl, Deighton, & Traue, 2002). The FEEL is a computerized test that measures the ability to recognize facially expressed emotions (Matsumoto & Ekman, 1988). Participants see pictures of facially expressed emotions on a computer screen and need to pick the right emotion out of six choices (surprise, happiness, disgust, sadness, fear and anger) within 10 s. The number of correct answers was chosen for the statistical analyses. The Feel tasks yields a Cronbach alpha of 0.76.

#### Executive functions

The Regensburg Word Fluency Test (RWT) (Aschenbrenner, Tucha, & Lange, 2000) was chosen to measure executive functions in terms of verbal fluency and verbal flexibility. Verbal fluency was measured with two subtests whereby for each subtest, participants had 2 min time to verbally produce as many words as they can, namely words starting with the letter “p” (phonemic category fluency subtest) as well as animals (semantic category fluency subtest). Verbal flexibility was also measured with two subtests whereby for each subtest, participants were told two categories and within 2 min had to produce alternately words of each category (word category 1, word category 2, word category 1…). In this way, they had to produce as many words as possible starting with the letters “h” and “t” (phonemic category flexibility subtest) as well as “sports” and “fruits” (semantic category flexibility subtest). The number of correctly produced terms of each of the four subtests was chosen for the statistical analyses. The RWT yields test–retest reliabilities of 0.76 (“p”-words), 0.77 (“h”-and “t”-words), 0.72 (“sports” and “fruits”), and 0.85 (“animals”).

#### Attention

Attention was measured using Test d2-Revision (d2-R; Brickenkamp, Schmidt-Atzert, & Liepmann, 2010), a neuropsychological measure of selective and sustained attention as well as visual scanning speed. In this test, the participants have 14 rows of symbols whereby they should only cross out the letters “d” that have two vertical dashes either above or below, or divided into one above and one below. They are asked not to cross out “d” with one, three, or four dashes as well as “p”, irrespective of the number of dashes. For each line, the participants have 20 s time whereby the examiner tells the participants when to change to the next line. The number of correctly crossed out “ds” was used for the statistical analyses. This task yields a Cronbach Alpha of 0.96.

#### Working memory

Working memory was assessed with the subtest Digit Span of the Wechsler Adult Intelligence Scale III (Aster von, Neubauer, & Horn, 2006). The subtest score is composed of two subcomponents, namely digit span forward and digit span backward. In both conditions, the participant is verbally presented with a string of digits and is asked to repeat the digits immediately upon hearing them. In the forward condition, participants are asked to repeat up to nine digits (depending on the performance) in exactly the same order as the digits were presented. In the backward condition, participants are asked to repeat up to eight digits (also depending on the performance) in reverse order. For each correctly repeated digit string, participants received one point. The number of correct repeat trials was used for the statistical analyses. This task yields a Split-Half reliability of 0.78.

### Procedure

Following domains were examined in group settings within each individual class: cognitive ToM, affective ToM, cognitive intelligence, and attention. The other domains were examined in single settings in free periods between lessons.

### Statistical analysis

Five analyses of variance (ANOVA) with age group (3) × gender (2) as independent variables and cognitive ToM (total score, 1st order, 2nd order, and 3rd order separately) as well as affective ToM as dependent variables including Bonferroni post hoc comparisons will be performed using SPSS.

In order to predict ToM ability across the whole age range, two regression analyses (enter method) will be conducted with cognitive ToM and affective ToM as dependent variables and age, cognitive and affective intelligence scores, attention, working memory, executive functions, and language comprehension as independent variables. Each independent variable of these two regression analyses (except for age itself) was previously age-adjusted by conducting separate regression analyses with age as independent variable and using the residuals for the current analyses.

In order to predict ToM ability within meaningful age groups, regression analyses will be performed regarding those age groups for which significant differences in ToM processing will be shown in the ANOVAs. Regression analyses will be performed in the respective age groups with cognitive ToM and affective ToM as dependent variables and cognitive and affective intelligence scores, attention, working memory, executive functions, and language comprehension as independent variables. As analyses will be conducted within age-groups, independent variables will not be age-adjusted.

The alpha value was set at 0.05 for all analyses. Sample size (power 1 − *β* = 0.99) was calculated using G*Power (Faul, Erdfelder, Lang, & Buchner, 2007).

## Results

### Cognitive ToM

The ANOVA regarding cognitive ToM total score (H1.1) showed significant main effects of *gender, F*(5,637) = 4.49, *η*_*p*_^2^ = 0.01, *p* = 0.035, and *age group, F*(5,637) = 26.23, *η*_*p*_^2^ = 0.08, *p* ≤ 0.0001, whereas no significant interaction effect between *gender* and *age group,* *F*(5,637) = 0.97, *η*_*p*_^2^ = 0.00, *p* = 0.380, could be found. Results showed that boys (*M* = 5.27, SD = 1.97) exhibited significantly lower cognitive ToM scores than girls (*M* = 5.64, SD = 2.06). Post hoc analyses showed that 13- to 14-year-olds scored significantly lower than 15- to 16-year-olds, *p* ≤ 0.0001, and 17- to 18-year-olds, *p* ≤ 0.0001. There was no significant difference between 15- to 16-year-olds and 17- to 18-year-olds, *p *= 0.064. For an overview, see Table 1.

The ANOVAs with cognitive 1st, 2nd and 3rd order ToM as dependent variables (H1.1) showed significant main effects of *age group* in all three orders (cognitive ToM 1st order, *F*(5,637) = 7.50, *η*_*p*_^2^ = 0.02, *p* = 0.001; cognitive ToM 2nd order, *F*(5,637) = 17.90, *η*_*p*_^2^ = 0.05, *p* ≤ 0.0001; and cognitive ToM 3rd order, *F*(5,637) = 18.71, *η*_*p*_^2^ = 0.06, *p* ≤ 0.0001. No significant effect of *gender* (cognitive ToM 1st order, *F*(5,637) = 2.55, *η*_*p*_^2^ = 0.00, *p* = 0.111; cognitive ToM 2nd order, *F*(5,637) = 2.00, *η*_*p*_^2^ = 0.00, *p* = 0.157; and cognitive ToM 3rd order, *F*(5,637) = 3.06, *η*_*p*_^2^ = 0.01, *p* = 0.081) or interaction effect between *gender* and *age group* (cognitive ToM 1st order, *F*(5,637) = 0.05, *η*_*p*_^2^ = 0.00, *p* = 0.946; cognitive ToM 2nd order, *F*(5,637) = 0.95, *η*_*p*_^2^ = 0.00, *p* = 0.387; and cognitive ToM 3rd order, *F*(5,637) = 0.97, *η*_*p*_^2^ = 0.00, *p* = 0.381) could be found. Post hoc comparisons regarding cognitive ToM 1st order revealed that the 13- to 14-year-olds reached significantly lower scores than the 17- to 18-year-olds, *p* ≤ 0.0001, but no lower scores than 15- to 16-year-olds, *p *= 0.272. No differences between 15- to 16-year-olds and 17- to 18-year-olds could be found, *p *= 0.116. Analyses regarding cognitive ToM 2nd order and cognitive ToM 3rd order showed that 13- to 14-year-olds reached significantly lower scores than 15- to 16-year-olds (cognitive ToM 2nd order, *p *= 0.002; cognitive ToM 3rd order, *p* ≤ 0.0001) and 17- to 18-year-olds (cognitive ToM 2nd order, *p* ≤ 0.0001; cognitive ToM 3rd order, *p* ≤ 0.0001. No significant differences could be found between 15- to 16-year-olds and 17- to 18-year-olds (cognitive ToM 2nd order, *p *= 0.051; cognitive ToM 3rd order, *p *= 0.999 ). For an overview, see Table 1.

### Affective ToM

With respect to affective ToM (H1.2), the ANOVA showed a significant main effect of *age group*, *F*(5,637) = 27.26, *η*_*p*_^2^ = 0.08, *p* ≤ 0.0001, but not of *gender*, *F*(5,637) = 1.86, *η*_*p*_^2^ = 0.00, *p* = 0.173. No significant interaction effect between *gender* and *age group*, *F*(5,637) = 2.88, *η*_*p*_^2^ = 0.01, *p* = 0.057, could be found. Post hoc comparisons regarding *age group* revealed similar results as above (13- to 14-year-olds vs. 15- to 16-year-olds, *p* ≤ 0.0001, and 17- to 18-year-olds, *p* ≤ 0.0001; 15- to 16-year-olds vs. 17- to 18-year-olds, *p *= 0.075). For an overview, see Table 1.

### Predictors of cognitive ToM

#### Whole sample

**Fig. 1:**
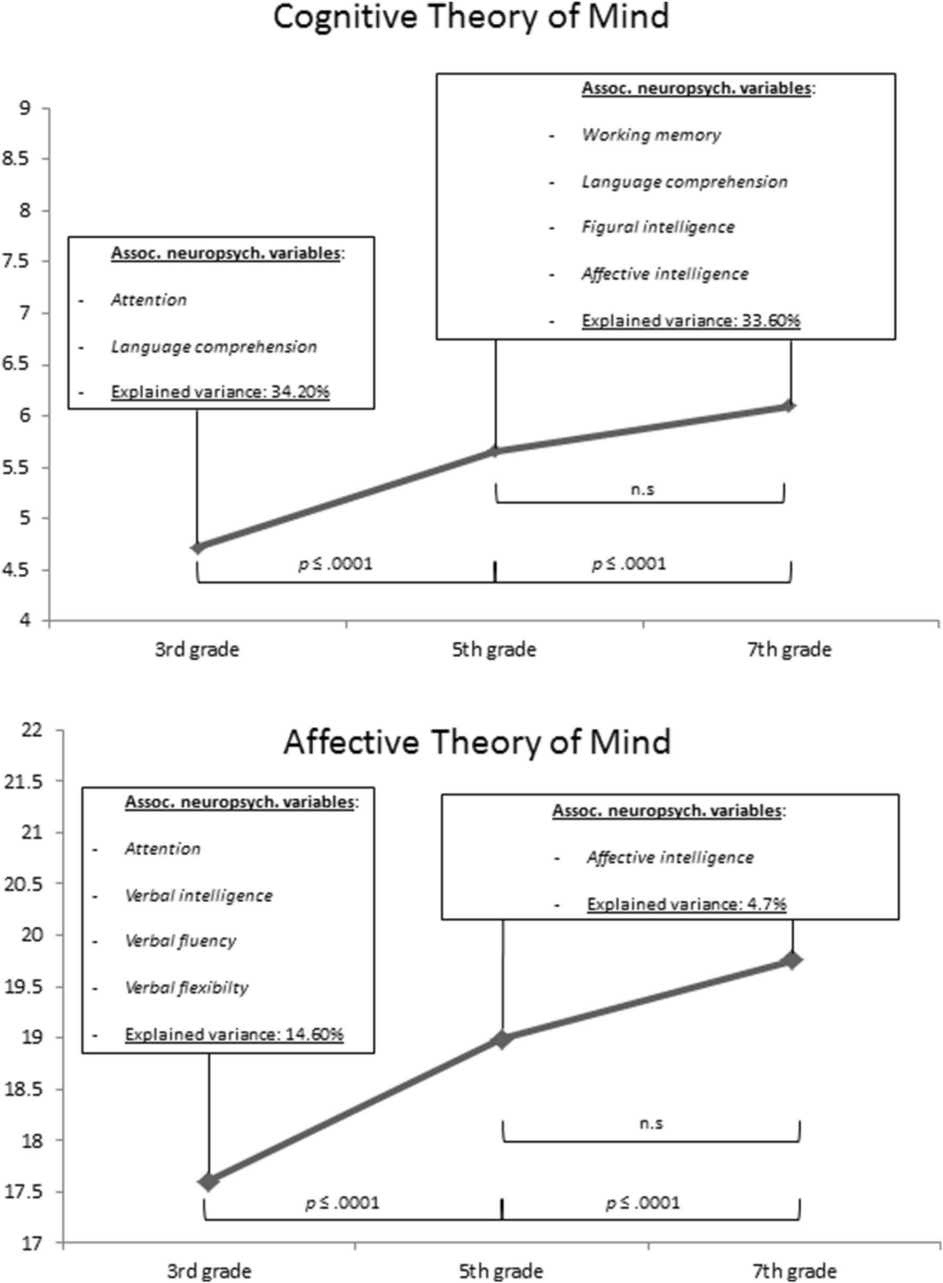
Differences between age groups regarding cognitive ToM (Theory of Mind) total score (upper panel) and affective ToM total score (lower panel) are shown. Associated neuropsychological variables and amount of explained variance by these variables are shown regarding the respective ToM scores for the youngest age group (3rd grade, 13–14 years) who showed the significantly lowest ToM values as well as combined for the older age groups (5th and 7th grade, 15–16 and 17–18 years) who did not differ significantly regarding ToM processing

Across all age groups (H1.3), the regression analysis showed a number of variables significantly predicting cognitive ToM performance (cognitive ToM total score), *F*(11,639) = 33.876, *p* ≤ 0.0001. 37.20% (*R*^*2*^ = 0.372) of the variation is accounted for by the predictors *age, p* ≤ 0.0001, *B* = 0.239, *β* = 0.222, *attention*, *p* ≤ 0.0001, *B* = 0.008, *β* = 0.123, *working memory*, *p* = 0.019, *B* = 0.052, *β* = 0.077, *figural intelligence*, *p* = 0.010, *B* = 0.060, *β* = 0.092, *affective intelligence*, *p* = 0.003, *B* = 0.044, *β* = 0.097, and *language comprehension*, *p* ≤ 0.0001, *B* = 0.250, *β* = 0.431. The predictors *phonemic category fluency* (RWT P), *p* = 0.761, *phonemic category flexibility* (RWT HT), *p* = 0.114, *semantic category fluency* (RWT Animals), *p* = 0.351, *semantic category flexibility* (RWT Sport-Fruits), *p* = 0.924 as well as *verbal intelligence*, *p* = 0.175, had no significant effects on cognitive ToM performance. *Numerical intelligence* was excluded from further analyses due to collinearity. Please see Fig. 1.

#### Predictors in 13- to 14-year-olds (3rd grade)

Within 3rd grade (H1.4), the regression analysis showed a number of variables significantly predicting cognitive ToM performance (cognitive ToM total score), *F*(11,215) = 9.619, *p* ≤ 0.0001. 34.20% (*R*^*2*^ = 0.342) of the variation is accounted for by the predictors *attention*, *p* = 0.002, *B* = 0.013, *β* = 0.192, and *language comprehension*, *p* ≤ 0.0001, *B* = 0.273, *β* = 0.472. The predictors *working memory*, *p* = 0.207, *phonemic category fluency* (RWT P), *p* = 0.254, *phonemic category flexibility* (RWT HT), *p* = 0.353, *semantic category fluency* (RWT Animals), *p* = 0.389, *semantic category flexibility* (RWT Sport-Fruits), *p* = 0.147, *affective intelligence*, *p* = 0.082 as well as *figural intelligence*, *p* = 0.169, *verbal intelligence*, *p* = 0.327, and *numerical intelligence*, *p* = 0.842 had no significant effects on cognitive ToM performance. Please see Fig. 1.

#### Predictors in 15- to 16-year-olds and 17- to 18-year-olds (5th and 7th grade)

In the combined group 5th and 7th grade (H1.5 and H1.6), the regression analysis showed a number of variables significantly predicting cognitive ToM performance (cognitive ToM total score), *F*(11,423) = 18.930, *p* ≤ 0.0001. 33.60% (*R*^2^ = 0.336) of the variation is accounted for by the predictors *working memory*, *p* = 0.041, *B* = 0.056, *β* = 0.088, *figural intelligence*, *p* = 0.025, *B* = 0.065, *β* = 0.107, *affective intelligence*, *p* = 0.044, *B* = 0.038, *β* = 0.085, and *language comprehension*, *p* ≤ 0.0001, *B* = 0.244, *β* = 0.443. Whilst the predictor *attention*, *p* = 0.061 shows a statistical trend, the predictors *phonemic category fluency* (RWT P), *p* = 0.279, *phonemic category flexibility* (RWT HT), *p* = 0.266, *semantic category fluency* (RWT Animals), *p* = 0.086, *semantic category flexibility* (RWT Sport-Fruits), *p* = 0.338 as well as *verbal intelligence*, *p* = 0.270, and *numerical intelligence*, *p* = 0.330 had no significant effects on cognitive ToM performance. Please see Fig. 1.

### Predictors of affective ToM

#### Whole sample

Across all age groups (H1.7), the regression analysis showed a number of variables significantly predicting affective ToM performance, *F*(11,639) = 7.247, *p* ≤ 0.0001. 11.30% (*R*^*2*^ = 0.113) of the variation in the outcome is accounted for by the predictors *age*, *p* ≤ 0.0001, *B* = 0.433, *β* = 0.250, a*ttention*, *p* = 0.044, *B* = 0.009, *β* = 0.083, and *affective intelligence*, *p* = 0.005, *B* = 0.080, *β* = 0.109. The predictors *working memory*, *p* = 0.377, *verbal intelligence*, *p* = 0.123, *figural intelligence*, *p* = 0.861, *phonemic category fluency* (RWT P), *p* = 0.213, *phonemic category flexibility* (RWT HT), *p* = 0.721, *semantic category fluency* (RWT Animals), *p* = 0.622, *semantic category flexibility* (RWT Sport-Fruits), *p* = 0.295, and *language comprehension*, *p* = 0.679 had no significant effects on affective ToM performance. *Numerical intelligence* was excluded from further analyses due to collinearity. Please see Fig. 1.

#### Predictors in 13- to 14-year-olds (3rd grade)

Within 3rd grade (H1.8), the regression analysis showed a number of variables significantly predicting affective ToM performance, *F*(11,215) = 3.160, *p* = 0.001. 14.60% (*R*^2^ = 0.146) of the variation in the outcome is accounted for by the predictors a*ttention*, *p* = 0.020, *B* = 0.019, *β* = 0.164, *verbal intelligence*, *p* = 0.031, *B* = 0.159, *β* = 0.160, *semantic category fluency* (RWT Animals), *p* = 0.005, *B* = -0.178, *β* = -0.233, and *semantic category flexibility* (RWT Sport-Fruits), *p* = 0.024, *B* = 0.477, *β* = 0.179. Whilst the predictor *affective intelligence*, *p* = 0.066 shows a statistical trend, the predictors *phonemic category fluency* (RWT P), *p* = 0.848, *phonemic category flexibility* (RWT HT), *p* = 0.093, *working memory*, *p* = 0.830, *language comprehension*, *p* = 0.185 as well as *numerical intelligence*, *p* = 0.074, and *figural intelligence*, *p* = 0.732 had no significant effects on affective ToM performance. Please see Fig. 1.

#### Predictors in 15- to 16-year-olds and 17- to 18-year-olds (5th and 7th grade)

In the combined group 5th and 7th grade (H1.9 and H1.10), the regression analysis showed that one variable significantly predicted affective ToM performance, *F*(11,423) = 1.830, *p* = 0.047. 4.7% (*R*^*2*^ = 0.047) of the variation in the outcome is accounted for by the predictor *affective intelligence*, *p* = 0.048, *B* = 0.068, *β* = 0.100. The predictors a*ttention*, *p* = 0.461, *working memory*, *p* = 0.146, *language comprehension*, *p* = 0.985, *phonemic category fluency* (RWT P, *p* = 0.098), *phonemic category flexibility* (RWT HT, *p* = 0.573), *semantic category fluency* (RWT Animals, *p* = 224), and *semantic category flexibility* (RWT Sport-Fruits, *p* = 0.855) as well as *verbal intelligence*, *p* = 0.506, *numerical intelligence*, *p* = 0.578, and *figural intelligence*, *p* = 0.683 had no significant effects on affective ToM performance. Please see Fig. 1.

## Discussion

The current study investigated cognitive and affective Theory of Mind (ToM) in early, middle, and late adolescence whilst additionally focusing on possible gender differences. This age span should be of special interest with respect to ToM development due to general neurodevelopmental changes in this period (e.g., Brain Development Cooperative Group, 2012; Lebel & Beaulieu, 2011; Shaw et al., 2008) as well as in conjunction with neurobiological correlates of ToM processing (e.g., Abu-Akel & Shamay-Tsoory, 2011). Nevertheless, research regarding ToM in adolescence is still scarce. The current study further aimed to create new knowledge by providing data on basic and higher order cognitive ToM processing in adolescence. Finally, based on previous behavioral and neuroscientific studies, this study investigated possible associations between neuropsychological variables and cognitive and affective ToM in adolescence within a single study design using a big sample.

### Development of cognitive and affective ToM: the role of age, gender, and complexity of ToM tasks

Group comparisons showed significant differences between the youngest adolescence group (13- to 14-year-olds) and the elder adolescence groups (15- to 16-year-olds and 17- to 18-year-olds) with respect to cognitive and affective ToM total scores. Results indicate a prominent increase between ages 13–14 and 15–16 regarding both types of ToM whilst no significant increase was shown between ages 15–16 and 17–18. This developmental step is supported by neurodevelopmental studies which show great changes regarding cortical gray and white matter volumes (e.g., Brain Development Cooperative Group, 2012), white matter tracts (Lebel & Beaulieu, 2011), cortical thickness (Shaw et al., 2008) and functional brain connectivity (e.g., Sato et al., 2014) in the form of a shift from local networks to more distributed networks and an increased number of connections within these network hubs (Sato et al., 2014).

Furthermore, literature shows specific changes regarding the whole brain as well as specific brain regions with respect to the age groups investigated in this study (e.g., Brain Development Cooperative Group, 2012; Krogsrud et al., 2014; Hu et al., 2013). In this context, it was shown that whole brain gray matter volumes decrease drastically until children reach approx. 15 or 16 years with a subsequent slower decrease (e.g., Brain Development Cooperative Group, 2012), indicating a great synaptic reorganization (e.g., Blakemore, 2008), presumably due to synaptic pruning processes (e.g., Huttenlocher, 1994). Furthermore, whole brain white matter volume (Brain Development Cooperative Group, 2012) and gray matter volume in different hippocampal subfields (e.g., Krogsrud et al., 2014) increase until children are approx. 15 with a subsequent slower increase. These white matter changes contribute to the synaptic reorganization in this age span (e.g., Jetha & Segalowitz, 2012) and are presumably induced by proceeding myelination and an increase in axon diameter (e.g., De Bellis et al., 2001; Perrin et al., 2008). Additionally, peaks in temporal gray matter and superior temporal lobe cortical thickness can be seen in ages 15–16 (Shaw et al., 2008) whereas a greater synaptic reorganization in the cingulate cortex presumably starts when adolescents approx. reach age 13 (Shaw et al., 2008). Besides great changes in subcortical gray matter volume between ages 14 and 16 (Brain Development Cooperative Group, 2012), it was shown that girls reach an amygdala peak volume at approx. 14 years with a slight decrease afterwards whereas boys show an ongoing rapid increase in amygdala volume until age 12 with a slowing increase afterwards (Hu et al., 2013).

These ongoing structural and functional changes in the adolescent brain as well as the specific neuronal changes within these age groups support the results regarding ToM development shown in the current study and comply with neurobiological models of ToM processing (e.g., Abu-Akel & Shamay-Tsoory, 2011). In this way, the developmental step in ToM processing shown in this study can be associated with ongoing changes in ToM-specific brain regions like for example the protracted development of the prefrontal cortex (PFC), specifically the medial PFC (e.g., Abu-Akel & Shamay-Tsoory, 2011; Blakemore, 2008, Konrad et al., 2013), and age-specific maturation of the cingulate cortex (e.g., Abu-Akel & Shamay-Tsoory, 2011; Schlaffke et al., 2015; Shaw et al., 2008), temporal regions (e.g., Abu-Akel & Shamay-Tsoory, 2011; Blakemore, 2008; Shaw et al., 2008), subcortical regions (Brain Development Cooperative Group, 2012), and the amygdala (Hu et al., 2013). Furthermore, it can be associated with changes in general brain connectivity (e.g., Sato et al., 2014) and ToM-specific connectivity between prefrontal, temporal, and temporo-parietal regions (Blakemore, 2008). These changes in ToM-specific regions and networks can be further related to the associated neuropsychological variables shown in the current study, as will be discussed later. Besides these structural and functional developments, great changes with respect to the serotonergic as well as the dopaminergic system occur throughout adolescence (see e.g., Steinberg, 2016) whereas these neurotransmitters were shown to greatly influence ToM processing (see e.g., Abu-Akel & Shamay-Tsoory, 2011).

With respect to basic cognitive ToM (1st order), the current study showed an age-related increase across adolescence whereas only 13- to 14-year-olds and 17- to 18-year-olds differed significantly. This result is somewhat surprising, given the low level of complexity in this order (see also e.g., Wimmer & Perner, 1983). This result could be possibly explained by differences in the allocation of attentional resources in the form of younger adolescents being overhasty in answering these easy questions without questioning their first choice. With respect to higher order ToM (2nd and 3rd order), a developmental step between ages 13–14 and 15–16 was shown. Whereas second-order ToM has already been thoroughly investigated (e.g., Brune & Brune-Cohrs, 2006; Perner & Wimmer, 1985), research on even more complex orders is still scarce, leaving unknown the developmental course of third-order ToM in adolescence. In this context, the current paper showed that the development of second- and third-order ToM is very similar across adolescence whereby these results get support from the previously mentioned neurodevelopmental changes in this age span.

With respect to affective ToM, no gender differences could be found. These results are consistent with previous behavioral findings (Vetter et al., 2013) which suggest that affective ToM is strongly influenced by age. This result could be possibly explained by converging levels of amygdala re-organization across adolescence (see e.g., Hu et al., 2013). In this context, Connolly, Lefevre, Young and Lewis (2018) showed only modest as well as very specific differences in emotion recognition between males and females.

Regarding cognitive ToM, female participants showed superior performance. Besides gender differences regarding structural brain development (Blakemore, 2008), functional differences regarding cognitive ToM could be found in the form of greater activation in the left mPFC and greater deactivation in the vmPFC/orbitofrontal cortex (OFC) in females (Frank et al., 2015) which possibly explains these differences. These differences could possibly be further explained by lasting effects of female children’s play behavior as it potentially promotes verbal communication abilities (Devine & Hughes, 2013). This would be supported by another result of the current study as it was shown that language comprehension was significantly correlated with cognitive ToM. Increased language comprehension ability potentially provides participants with greater resources to represent and communicate about mistaken beliefs (Milligan, Astington, & Dack, 2007).

### Neuropsychological variables associated with cognitive and affective ToM

In this study, the regression analyses showed that age was significantly associated with both cognitive and affective ToM processing across adolescence. This result further highlights the importance of age regarding ToM performance and is supported by studies on neurodevelopment in this age span (as discussed above). Besides age, a number of neuropsychological abilities were shown to be associated with ToM processing across adolescence whereby for these analyses age-adjusted (the effect of age was extracted by preceding analyses) values were used.

In this context, it was shown that affective intelligence and attention were significantly correlated with cognitive and affective ToM in adolescence. This result is supported by studies which show a bidirectional influence of many cognitive and emotional processes (e.g., Okon-Singer, Hendler, Pessoa, & Shackman, 2015), whereby regarding this influence, shared underlying neural networks were shown (e.g., Okon-Singer et al., 2015; Pessoa, 2008). Key regions and hubs for this cognition–emotion integration are for example the prefrontal cortex and the amygdala (e.g., Pessoa, 2008) which are central regions in the ToM network (e.g., Abu-Akel & Shamay-Tsoory, 2011). In this context, in their neurobiological model, Abu-Akel and Shamay-Tsoory (2011) show that cognitive and affective aspects of ToM processing rely on overlapping and linked brain regions which further supports the results of the current study.

With respect to affective ToM, the association with affective intelligence in terms of recognizing and understanding emotions was not surprising as it is in line with the definition of Mayer and Salovey (1997). Regarding cognitive ToM, the association with affective intelligence is less obvious. Nevertheless, on closer inspection of the cognitive ToM paradigm used in this study (a false belief task), several connecting factors for affective intelligence can be seen. While performing the cognitive ToM task, the participants witness a lot of false beliefs as well as erroneous actions and decisions done either by persons that represent themselves in the role of children in a family or by other important people in an adolescent’s family life like parents or siblings. One explanation could therefore be that children with a greater affective intelligence are more able to empathize with the protagonists and to understand the feelings that result from such mistakes, and therefore dedicate more attentional resources to the “rectification” of these situations, in the form of the answers given in the task. The result regarding affective intelligence is further in line with previous research that shows that affective intelligence seemingly develops before mental states are understood and predicts ToM at a later age (Mier et al., 2010; O’Brien et al., 2011). Given this developmental aspect as well as research on general or ToM-specific integration of cognitive and affective processes, it can be hypothesized that individuals who are aware of their own and other people’s emotions are more alert to social cues and therefore more likely to notice discrepancies between their own and other’s experiences.

In this study, attention in the form of selective attention and response inhibition were significantly correlated with affective and cognitive ToM performance. It is not surprising that the ability to continuously focus on relevant stimuli whilst inhibiting distracting irrelevant stimuli (see e.g., Koziol et al., 2014) facilitates the formation of basic and higher order mental constructs like ToM. Furthermore, research increasingly indicates a strong link between attentional processes and the processing of emotional stimuli (e.g., Okon-Singer et al., 2015). In this context, it can be seen that attention networks in the brain which still evolve throughout adolescence (for an overview see e.g., Koziol et al., 2014) share regions with the ToM network (see e.g., Abu-Akel & Shamay-Tsoory, 2011). In their neurobiological model, Abu-Akel and Shamay-Tsoory (2011) specifically note that the relevance of stimuli to self or other mental states is assigned through the dorsal as well as the ventral attention system. In this context, white matter maturation was shown to be associated with increases in attentional resources (for an overview, see e.g., Jetha & Segalowitz, 2012).

Whilst affective ToM was exclusively associated with the neuropsychological variables attention and affective intelligence, it was shown that cognitive ToM performance in adolescence also correlated with working memory, figural intelligence, and language comprehension. This result could be due to methodological reasons as the affective ToM task requires inferring affective states on basis of given pictorial stimuli, whereas the cognitive ToM task measures basic and higher order ToM requiring to build complex mental constructs, as will be discussed below.

The cognitive ToM measure that was used in the current study seemingly presents all relevant information the whole time a story is executed. As working memory is associated with cognitive ToM processing, it could be hypothesized that performance depends on the willingness to take up and update the information in an adequate way. In this way, weak ToM performance could be explained by overestimating one’s own memory performance and not use the presented information successfully like, e.g., going back in the story to re-analyze information (see e.g., meta-memory, Dunlosky & Thiede, 2013; the memory illusion phenomena, Chabris & Simons, 2010; Shaw, 2016). Another logic explanation would be that ToM performance does not only depend on the willingness to process information adequately, but additionally on the capacity to keep information in mind and to process it. In order to process basic and higher order cognitive ToM, one needs to take up information, process it, and to continuously update it so as to produce a mental image and to keep the current status in mind. In this way, higher order ToM requires to use more information to impute mental states to oneself or others (e.g., X knows that Y knows that Z does not know) than basic ToM and therefore requires higher working memory capacity involving the ability to inhibit uneconomic processing of irrelevant information. This would be supported by an ongoing maturation of the hippocampus (Krogsrud et al., 2014), the prefrontal cortex (see e.g., Blakemore, 2008; D’Esposito & Postle, 2014) and whole brain white matter (e.g., Jetha & Segalowitz, 2012), and would be in line with information processing procedures as depicted in the “Predication Model” by Kintsch (2001).

Another neuropsychological variable that was associated with cognitive ToM processing is figural intelligence in the form of nonverbal, fluid reasoning (see e.g., Amthauer et al., 2001; Flanagan & Kaufman, 2009; Wechsler, 2014). This result is supported by shared neural brain regions between reasoning and ToM like, for example the prefrontal cortex (e.g., Abu-Akel & Shamay-Tsoory, 2011; Donoso et al., 2014), the developmental aspects of this region (e.g., Blakemore, 2008) as well as white matter maturation (e.g., Jetha & Segalowitz, 2012). This factor further measures processing of figural material, building logical relations between single aspects and the whole, simultaneous processing, understanding proportions, as well as classification ability (see e.g., Amthauer et al., 2001; Flanagan & Kaufman, 2009; Wechsler, 2014). These aspects could facilitate cognitive ToM processing as similar approaches have to be taken to solve a problem (e.g., analysis of structured information, comparison of solutions, inclusion or exclusion of solutions, coming to a clear conclusion) as well as by facilitating these processes by enabling visual–spatial mentalization of the described actions in the stories. In this context, a close link between visual perspective taking and ToM performance was previously shown as both tasks require understanding and switching of perspective as well as show shared neuronal activation (see e.g., Schurz et al., 2013).

The last significant neuropsychological variable correlated with cognitive ToM is language comprehension. This result is not surprising since verbally presented contents need to be understood before mental constructs can be built and the right multiple-choice answer can be chosen. This result is supported by previous behavioral studies (e.g., Ahmed & Miller, 2011; Astington & Jenkins, 1999; Frank et al., 2015) as well as by studies showing shared neural brain regions between language processing and ToM like for example the temporal lobes (e.g., Abu-Akel & Shamay-Tsoory, 2011; Szaflarski et al., 2012), its neurodevelopmental aspects (e.g., Shaw et al., 2008) as well as white matter maturation (e.g., Jetha & Segalowitz, 2012).

The regression analyses within age groups in the current study give a more detailed view on the relation between ToM processing and neuropsychological abilities in different parts of adolescence. As preceding results of the current study showed that 13- to 14-year-olds exhibit significantly lower ToM scores than 15- to 16- and 17- to 18-year-olds, and that the later age groups did not differ, regression analyses were performed in age group 13–14 years and a combined age group (15–16 and 17–18 years). As analyses were performed within distinct age groups, the neuropsychological variables were not age-adjusted.

The results yield that the separate age groups show (mainly) different associated neuropsychological variables, whereas the age-specific increase or decrease regarding the number of predicting variables differs between cognitive and affective ToM (see Fig. 1). In this context, it can be seen that the neuropsychological variables attention and affective intelligence which were previously shown to be associated with both cognitive and affective ToM across adolescence, show differences between age groups.

In 13- to 14-year-olds, attention was shown to be predictive of both cognitive and affective ToM whilst this effect was not shown in the older age groups. In this context, it can be hypothesized that younger adolescents need to focus their cognitive resources more heavily on processing relevant stimuli and inhibiting distracting stimuli (see, e.g., Koziol et al., 2014) whilst this effort presumably decreases with age. This interpretation would be supported by studies on still evolving attention and ToM networks in the adolescent brain (see, e.g., Abu-Akel & Shamay-Tsoory, 2011; Jetha & Segalowitz, 2012; Koziol et al., 2014).

Cognitive ToM in 13- to 14-year-olds was further associated with language comprehension. In this context, it can be hypothesized that young adolescents additionally focus their cognitive resources on processing and understanding verbally presented contents. This would be supported by studies which show an association between verbal abilities like pragmatic language processing or syntax processing, and ToM performance (Astington & Jenkins, 1999; Frank et al., 2015). As younger adolescents show lower cognitive ToM scores than elder adolescents, it can be hypothesized that they show lower cognitive resources which limits their possibilities to process the information in a more efficient and logical way. This would be supported by the results of the elder adolescents group (see discussion below and Fig. 1).

Affective ToM performance in 13- to 14-year-olds was, additionally to its association with attention, further associated with verbal intelligence, verbal fluency, and verbal flexibility. It can therefore be hypothesized that at this age, adolescents who have a greater vocabulary, know more word meanings and attributions, and can compare these verbal contents more flexibly (for properties of the tasks see, e.g., Amthauer et al., 2001; Aschenbrenner et al., 2000; Flanagan & Kaufman, 2009; Wechsler, 2014) show an advantage in processing the emotion words of the current affective ToM task. Nevertheless, given that affective intelligence was not associated with affective ToM in this age group, the results indicate that younger adolescents show a greater variability in their ability to integrate different emotional stimuli (pictures, words) into a representation of another person’s mental state. This interpretation would be supported by studies on affective intelligence development across childhood and adolescence (see, e.g., Williams et al., 2009).

In the combined group (15–16 and 17–18 years), affective intelligence was shown to be predictive of both cognitive and affective ToM. In this context, it can be hypothesized that elder adolescents are increasingly able to empathize with other individuals with regard to feelings that arise from mistakes and as a consequence dedicate more attentional resources to distinguish between mental states of one self and others (cognitive ToM, false belief task). Based on studies on increasing affective intelligence across adolescence and its association with affective ToM (see, e.g., Mier et al., 2010; O’Brien et al., 2011; Williams et al., 2009), it can be further hypothesized that elder adolescents are more perceptive of social cues of one self and others and therefore show better affective ToM performances.

Cognitive ToM in the combined age group (15–16 and 17–18 years) was further associated with working memory, figural intelligence, and language comprehension. Similar to age group 13–14 years, also in the combined age group (15–16 and 17–18 years) an association between cognitive ToM and language comprehension was shown which indicates that the previously mentioned verbal abilities build a basis for ToM performance in the current task. Nevertheless, based on studies on general brain development (e.g., Blakemore, 2008; Brain Development Cooperative Group, 2012; Shaw et al., 2008), it can be hypothesized that greater cognitive resources enable elder adolescents to use additional cognitive abilities in the course of ToM processing. As the cognitive ToM task is clearly structured and requires clear structured processing procedures, it can be assumed that elder adolescents are increasingly capable of processing ToM-specific information. In this way, they could benefit from an increased motivation and/or capacity to take up and update information in an adequate way and to inhibit processing of irrelevant information. This would be supported by studies on hippocampus, PFC, and white matter maturation (Blakemore, 2008; D’Esposito & Postle, 2014; Jetha & Segalowitz, 2012; Krogsrud et al., 2014). Elder adolescents presumably benefit additionally from an increased logical thinking ability such as analyzing structured information, comparing as well as including or excluding solutions, or reaching a clear conclusion (for a aspects of figural intelligence in terms of nonverbal, fluid reasoning see, e.g., Amthauer et al., 2001; Flanagan & Kaufman, 2009; Wechsler, 2014).

Numerical intelligence was not shown to be associated with either cognitive or affective ToM. At least with respect to cognitive ToM, this is somehow surprising, given that the subtests measure the ability to detect logical relations between numbers involving reasoning processing procedures (see Amthauer et al., 2001) that would be suitable for the processing of cognitive ToM in this study, as discussed above. On the other hand, it requires numeracy involving crystalline knowledge about mathematical operations (see e.g., Amthauer et al., 2001). In this context, imaging studies suggest that children’s numeracy is strongly associated with activation in ToM-specific regions such as the prefrontal cortex, anterior cingulate cortex, and the hippocampus which indicates that children require more attentional as well as working memory resources (Rivera, Reiss, Eckert, & Menon, 2005). This pattern, though, changes across adolescence involving a decrease in activation in these regions (Rivera et al., 2005), indicating that in this age span numerical intelligence increasingly relies on other resources that are not shared with those regarding cognitive ToM processing. Another explanation could be, although not controlled for in this study, that mathematics anxiety mediated the performance on these subtests as studies show that even medium levels of mathematics anxiety influence numeracy significantly and negatively (e.g., Ashcraft & Faust, 1994).

## Conclusion

An age-related increase in basic affective and cognitive ToM across early, middle, and late adolescence as well as a developmental step regarding higher order cognitive ToM between age 13–14 and 15–16 years was shown. Girls outperformed boys regarding cognitive ToM. Across adolescence, increases in cognitive and affective ToM were associated with age and the neuropsychological variables attention and affective intelligence. Cognitive ToM performance across adolescence was further associated with working memory, language comprehension, and figural intelligence in the form of nonverbal fluid reasoning. Results also showed that in 13- to 14-year-olds cognitive and affective ToM performance is associated with attention. In this age group, cognitive ToM was further associated with language comprehension whereas affective ToM was associated with verbal intelligence, verbal fluency, and verbal flexibility. In 15–16- and 17- to 18-year-olds cognitive and affective ToM were associated with affective intelligence. In this age group, cognitive ToM was further associated with working memory, language comprehension, and figural intelligence. The results of the current study are well supported by studies on the neurobiological bases of ToM, the neuronal correlates of the neuropsychological variables, and the neuro-biologically supported integration of cognitive and affective processes. Ongoing maturation as well as specific changes in the brain in the investigated age span additionally supports the behavioral results shown in the current study.

## Data Availability

The dataset is freely available under the Open Science Framework (OSF) link (https://mfr.osf.io/render?url=https%3A%2F%2Fosf.io%2Fvbdpf%2Fdownload)—name: “Cognitive and affective ToM_Psychological Research_1.sav”.

## Appendix

Example of the Theory of Mind Stories for Children and Adolescents.

### The car key

The parents, Rosa and Paul, are driving home from work by car. After their arrival at home, they put the car key on a little table in the kitchen. While Rosa is reading a book in the garden, Paul drives to a pizzeria to buy something to eat for dinner. Back home, he puts the car key on a little table in the anteroom and takes the dog for a walk.

In the meantime, Rosa gets a call from her daughter who wants her to pick her up from her friend’s place. Rosa is going to get the car key.

## Footnotes

1 The neuroscientific discipline “Neuropsychology” addresses the association between measurable behavior and its anatomical, physiological, and biochemical cerebral bases (see e.g., Hartje & Poeck, 2006; Karnath & Thier, 2006). By using, inter alia, (neuro-)psychological tests, inter-individual differences can be measured with respect to neuropsychological domains such as perception, attention/concentration, memory, executive functions, language processing, (visuo-)construction performance, intellectual abilities, or affective processing (see, e.g., Hartje & Poeck, 2006; Karnath & Thier, 2006; Lezak, Howieson, Loring, & Fischer, 2004). For an overview of neuropsychological assessment tools please see, for example, Lezak et al. (2004) or Strauss, Sherman, and Spreen (2006). In the current study, specific subdomains of neuropsychological abilities were investigated, based on previous studies regarding the relation between Theory of Mind processing and cognitive variables.
